# Efficacy and safety of consolidation chemotherapy after adjuvant therapy in stage IB-IIA cervical cancer patients with risk factors: a retrospective single-center study

**DOI:** 10.3389/fonc.2024.1374195

**Published:** 2024-03-21

**Authors:** Jiaxin Wang, Huaijuan Guo, Jingjing Yang, Jingxian Mao, Ying Wang, Ruidong Gao, Xuebing Yan, Jie Wang

**Affiliations:** ^1^ Department of Oncology, The Affiliated Hospital of Yangzhou University, Yangzhou University, Yangzhou, China; ^2^ Department of Oncology, Baoying Traditional Chinese Medicine Hospital, Yangzhou University, Yangzhou, China

**Keywords:** cervical cancer, risk factor, chemoradiotherapy, consolidation chemotherapy, prognosis

## Abstract

**Objective:**

Accumulated evidence has suggested a relatively high recurrence rate in early-stage cervical cancer (CC) patients with risk factors. This study aimed to assess the efficacy and safety of consolidation chemotherapy following adjuvant therapy (concurrent chemoradiotherapy (CCRT) or radiotherapy (RT) alone) in stage IB-IIA CC patients with risk factors.

**Methods:**

A total of 237 stage IB-IIA CC patients who received radical surgery between January 2014 and December 2021 were included in the retrospective study. According to the types of adjuvant therapies, the patients were classified into the control group (CCRT or RT alone) and the study group (consolidation chemotherapy following CCRT or RT alone). The propensity score matching (PSM) was used to balance baseline characteristics between the two groups. The primary end points of the study were disease-free survival (DFS) and overall survival (OS).

**Results:**

For the entire cohort, no significant difference was observed in the DFS or OS between the study and control group, which was also confirmed in the PSM cohort (n=124). The multivariate analysis identified the high-risk factor type was an independent adverse prognostic factor for the patients. In patients with high risk factors, consolidation chemotherapy following adjuvant therapy was significantly associated with better clinical outcomes and identified as an independent prognostic favorable factor. Moreover, this association remained statistically significant in high-risk patients with ≥2 metastatic lymph nodes. In patients with intermediate risk factors, consolidation chemotherapy following adjuvant therapy was unrelated to DFS or OS. The safe assessment demonstrated consolidation chemotherapy following adjuvant therapy was significantly correlated with higher rates of ≥ grade 3 hematologic toxicities in both the global and subgroup analysis stratified by risk factor type.

**Conclusion:**

Consolidation chemotherapy after adjuvant therapy provided survival benefits in stage IB-IIA CC patients with high risk factors, particularly those with ≥2 metastatic lymph nodes. However, related hematologic toxicities should be alerted in patient management. The actual efficacy and safety of consolidation chemotherapy still need to be investigated in more well-designed clinical trials.

## 1 Introduction

Cervical cancer (CC) is the fourth most commonly diagnosed cancer in women, with approximately 604,000 new cases and 342,000 deaths worldwide in 2020 (1). More than half of CC cases and CC related deaths are estimated in Asian countries, where China is responsible for 18% and 17% respectively (2). More than 90% of CC is caused by human papillomavirus (HPV) infection, and therefore cervicovaginal HPV testing combined with vaccination against HPV is currently the most effective strategy for disease prevention (3). Radical hysterectomy with pelvic lymphadenectomy is the current standard treatment for early-stage cervical cancer based on the International Federation of Gynecology and Obstetrics (FIGO) staging system. After radical surgery, adjuvant radiation with concurrent cisplatin-based chemotherapy is performed for early-stage CC patients with high-risk factors including lymph node metastasis, parametrial invasion, and resection margin involvement (4). However, approximately 20% of patients will develop recurrence after concurrent radiochemotherapy, suggesting the necessity of more aggressive therapies for these patients (5). Furthermore, for patients with intermediate risk factors including lymphovascular space involvement (LVSI), large tumor size, and deep stromal invasion (DSI), adjuvant therapeutic strategies remain a topic of debate among medical centers and regions (6). Therefore, more clinical evidence is urgently needed to help clinicians make individualized therapy decision for early-stage CC patients.

A recent meta-analysis has demonstrated consolidation chemotherapy after concurrent chemoradiation could effectively reduce the local and distant recurrence rate in locally advanced CC (LACC) patients, raising a question whether it also benefits early-stage CC patients with risk-factors (7). A recent retrospective study has demonstrated consolidation chemotherapy following postoperative concurrent chemoradiotherapy (CCRT) could significantly improve the disease-free survival (DFS) and overall survival (OS) of early-stage CC patients with more than two positive lymph nodes or high-risk factors (8). However, other similar studies failed to prove its prognostic benefit in early-stage CC patients with risk-factors (9, 10). Moreover, consolidation chemotherapy after CCRT was found to associate with an increased grade 3/4 myelosuppression rate (11). Therefore, the actual impact of consolidation chemotherapy after CCRT or radiotherapy (RT) on early-stage CC patients with high or intermediate risk factors remains a controversial issue and a related clinical trial is ongoing (No. NCT00980954).

In this study, we hypothesized that consolidation chemotherapy after adjuvant therapy might be effective and safe in early-stage CC patients with risk factors. For verifying this hypothesis, a retrospective cohort enrolling 237 FIGO stage IB1-IIA2 patients with high or intermediate risk factors was utilized. The primary aim of this study was to investigate the clinical efficacy of consolidation chemotherapy after CCRT or RT, and the secondary aim was to evaluate its safety in stage IB-IIA CC patients.

## 2 Methods

### 2.1 Patient selection

**Figure 1:**
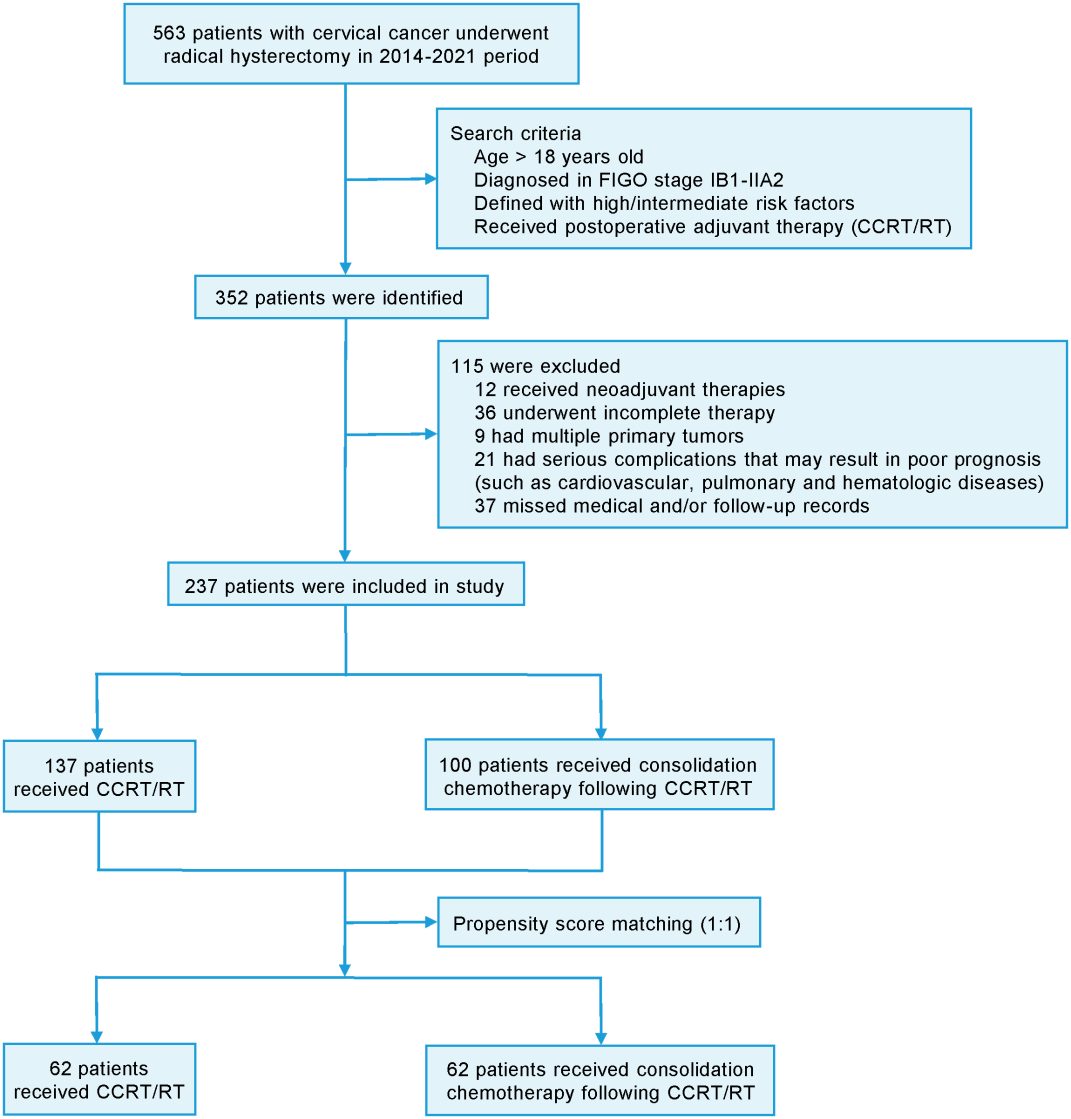
Flowchart of the patient recruitment in the retrospective cohort.

This single-center retrospective study was approved by the ethics committee of Affiliated Hospital of Yangzhou University (No.2022-YKL11) and informed consents were obtained from patients for using their information in scientific researches. The CC patients were selected at the oncology department of Affiliated Hospital of Yangzhou University between January 2014 and December 2021. The inclusion criteria were as follows: (1) age > 18 years; (2) patients were pathologically diagnosed as CC and received radical surgery; (3) IB1-IIA2 stage (2009 FIGO staging system); (4) presence of defined high or intermediate risk factors (12); (5) patients received postoperative adjuvant therapy (CCRT or RT) with or without consolidation chemotherapy. In detail, high risk factors included lymph node metastasis, parametrial invasion and positive resection margins. Intermediate-risk factors defined by the Sedlis criteria included as follows: (1) LVSI combined with deep one-third cervical stromal invasion and tumor of any size; (2) LVSI combined with middle one-third stromal invasion and tumor size ≥ 2 cm; (3) LVSI combined with superficial one-third stromal invasion and tumor size ≥ 5 cm; (4) no LVSI but deep or middle one-third stromal invasion and tumor size ≥ 4 cm. The definitions of these risk factors were ensured to be consistent in their application throughout the study. The exclusion criteria were as follows: (1) patients received neoadjuvant therapies; (2) incomplete therapy; (3) multiple primary tumors; (4) serious complications (such as cardiovascular, pulmonary and hematologic diseases) that may result in poor prognosis; (5) lack of medical and/or follow-up records. For minimizing the potential impact of baseline differences between the groups, the propensity score matching (PSM) was utilized with a ratio of 1:1. The flow chart of patient recruitment was demonstrated in **Figure 1**.

### 2.2 Treatment regimens

All the patients received the radical surgery performed by the same group of surgeons. Adjuvant pelvic radiation with 3-dimensional conformal RT or intensity-modulated RT (IMRT) was initiated within 4-6 weeks after surgery. Pelvic RT was given in 25-28 fractions for a total dose of 45.0 to 50.4 Gy. For concurrent chemotherapy, cisplatin (30-40 mg/m^2^) was administered weekly for three to five cycles (n=44) or paclitaxel (135 mg/m^2^)/nab-paclitaxel (260 mg/m^2^) combined with cisplatin (60 mg/m^2^) was administered every 3 weeks for two to three cycles (n=11) during RT. High-dose rate intracavitary brachytherapy was performed for patients with a close (<5 mm) or positive surgical margin of the vagina. The total dose was 15 Gy in three fractions and was delivered to a depth of 5 mm below the vaginal mucosa.

Consolidation chemotherapy was initiated approximately 3 weeks after the completion of radiation. The following chemotherapy regimens were performed every 3-4 weeks for four to six cycles: (1) paclitaxel (135-175 mg/m^2^) plus cisplatin (60-75 mg/m^2^)/carboplatin (AUC=5.0), (n=85); (2) nab-paclitaxel (260 mg/m^2^) plus cisplatin (60-75 mg/m^2^), (n=7); (3) doxorubicin liposome (30 mg/m^2^) plus cisplatin (60-75 mg/m^2^)/nedaplatin (80 mg/m^2^), (n=5); (4) etoposide (100 mg/m^2^/d*3d) plus cisplatin (60-75 mg/m^2^) (n=3). In case that 3/4 grade adverse events were observed, the chemotherapy doses were reduced by 20%. In case that the adverse events continued after the adverse events, the chemotherapy was stopped until the adverse events were resolved. The study group was defined as patients receiving consolidation chemotherapy following pelvic radiation or concurrent chemoradiotherapy, while the control group was defined as those only receiving pelvic radiation or concurrent chemoradiotherapy.

### 2.3 Follow-up and study endpoints

The follow-up examination was conducted every 3 months in the first two years, every 6 months from the third to fifth years, and annually after five years. The date of the last follow-up was December 30^th^ 2022. The primary endpoints of this study included disease-free survival (DFS), overall survival (OS) and treatment safety. DFS was defined as the time from surgery to the date of distant metastasis, local recurrence, or the last follow-up. OS was defined as the time from surgery to death from any cause or the last follow-up.

### 2.4 Statistical analysis

The statistical analyses were conducted using SPSS software (version 22.0, IBM, Armonk, NY, USA) and R package (version 4.3.0). Continuous variables were described as median values and compared using Mann-Whitney U test. The categorical variables were analyzed with the chi-square test or Fisher’s exact test. Survival curves were generated by the Kaplan-Meier method and compared using the log-rank test. Significant prognostic factors affecting DFS or OS were identified using the univariate and multivariate analysis based on Cox regression models. A two-tailed p value less than 0.05 was considered statistically significant.

## 3 Results

### 3.1 General description of patient characteristics

According to the inclusion and exclusion criteria, a total of 237 CC patients were finally included in the study and their clinicopathological characteristics were summarized in **Table 1**. In the entire cohort, 193 (81.4%) patients received laparotomic surgery, compared with 44 (18.6%) patients receiving laparoscopic surgery. 100 patients (42.2%) received additional consolidation chemotherapy after pelvic radiation or concurrent chemoradiotherapy. The patients underwent radical hysterectomy (Querleu-Morrow Type C) combined with pelvic lymph node dissection with or without para-aortic lymph node sampling. The median number of removed lymph nodes was 17 for both the study and control group. No significant difference was observed in the surgery types (laparotomic or laparoscopic surgery) between the study and control group (p=0.627). No significant difference was observed in the recurrence rate and type between the laparotomic and laparoscopic group (all p>0.05). No significant difference was observed in age, tumor differentiation, LVSI, DSI, number of pelvic lymph nodes retrieved and surgical margin involvement between the study and control group (all p>0.05). However, significant difference was observed in histologic type (p=0.001), FIGO stage (p=0.001), tumor size (p=0.017), the number of pelvic lymph node (LN) (p<0.001) and parametrial invasion (10.0% *vs.* 1.5%, p=0.003). 28 (11.8%) patients were dead during the follow-up period, among which 27 cases were cancer-specific. 37 (15.6%) patients experienced disease recurrence, where local recurrence alone, distant metastasis alone, and the both were observed in 12 (32.4%), 16 (43.2%), and 9 (24.3%) patients respectively. After PSM with a ratio of 1:1, a total of 124 patients were identified and their clinicopathological characteristics were also summarized in **Table 1**. The statistical analysis revealed no significant difference in their clinicopathological characteristics between the study and control group (all p>0.05).

**Table 1:** Baseline characteristics of the entire cohort before and after propensity score matching.

Characteristics	Before PSM		P value	After PSM		P value
	Control group (n=137, 100%)	Study group (n=100,100%)		Control group (n=62, 100%)	Study group (n=62, 100%)	
Age (years old)						
Median (IQR)	52 (47-59)	51 (45-57)	0.175	50(45-58.3)	51(47-58)	0.492
Histologic type						
Squamous	131 (95.6)	82 (82.0)	**0.001**	56(90.3)	58(93.5)	0.510
Non-squamous	6 (4.4)	18 (18.0)		6(9.7)	4(6.5)	
Differentiation						
Well + moderate	91 (66.4)	62 (62.0)	0.482	41(66.1)	45(72.6)	0.436
Poor	46 (33.6)	38 (38.0)		21(33.9)	17(27.4)	
FIGO stage						
IB	105 (76.6)	56 (56.0)	**0.001**	39(62.9)	39(62.9)	1.000
IIA	32 (23.4)	44 (44.0)		23(37.1)	23(37.1)	
Tumor size (mm)						
<40	91 (66.4)	51(51.0)	**0.017**	35(56.5)	34(54.8)	0.857
≥40	46 (33.6)	49 (49.0)		27(43.5)	28(45.2)	
LVSI	96 (70.1)	81 (81.0)	0.056	45(72.6)	44(71.0)	0.842
Deep stromal invasion	113 (82.5)	89 (89.0)	0.162	53(85.5)	54(87.1)	0.794
No. of pelvic LN retrieved						
Median (IQR)	17 (14-20)	17(13-24)	0.548	17(13-19)	16(11-24)	0.701
Pelvic LN metastasis	19 (13.9)	42 (42.0)	**<0.001**	18(29.0)	19(30.6)	0.844
Parametrial invasion	2 (1.5)	10 (10.0)	**0.003**	2(3.2)	2(3.2)	1.000
Surgical margin involvement	2 (1.5)	5 (5.0)	0.230	1(1.6)	1(1.6)	1.000

PSM, Propensity score matching; LVSI, lymphovascular space involvement; LN, lymph node. The bold values mean p value less than 0.05.

### 3.2 Efficacy of consolidation chemotherapy after adjuvant therapy in the entire cohort

**Figure 2:**
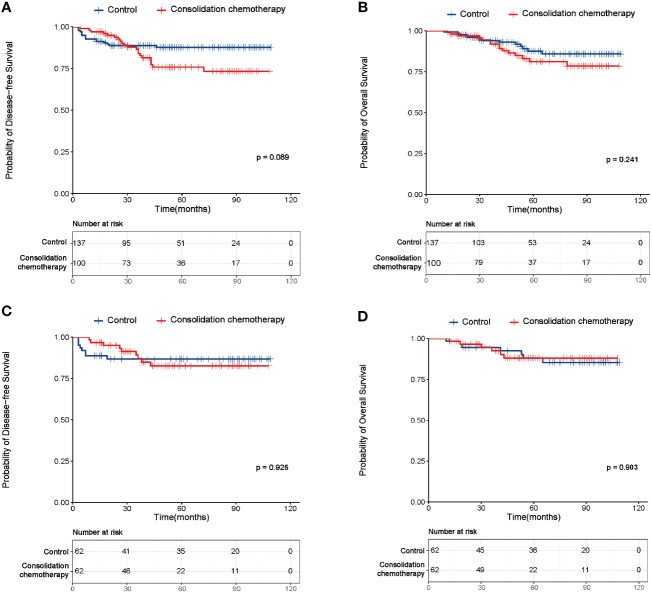
Survival curves of the study and control group of the entire cohort before and after propensity score matching (PSM). **(A)** Disease-free survival (DFS) curves of the study and control group of the entire cohort before PSM. **(B)** Overall survival (OS) curves of the study and control group of the entire cohort before PSM. **(C)** DFS curves of the study and control group of the entire cohort after PSM. **(D)** OS curves of the study and control group of the entire cohort after PSM.

For the entire cohort, before PSM, no significant difference was observed in the DFS and OS between the study and control group (DFS: p=0.089, **Figure 2A**; OS: p=0.241, **Figure 2B**). This finding was then confirmed in the cohort after PSM (DFS: p=0.925, **Figure 2C**; OS: p=0.903, **Figure 2D**). As shown in **Tables 2**, **3**, before PSM, the univariate analysis revealed FIGO stage and risk factor were significantly correlated with both DFS and OS, both of which were also identified as independent prognostic factors in the following multivariate analysis. After PSM, the univariate and multivariate analyses also indicated FIGO stage and risk factor were the independent predictive factors for DFS and OS (**Supplementary Tables 1**, **2**).

**Table 2:** Univariate and multivariate analysis for the disease-free survival of the entire cohort before propensity score matching.

Characteristics	Reference	Disease-free survival					
		Univariate analysis			Multivariate analysis		
		HR	95% CI	P	HR	95% CI	P
Histology	Squamous	2.227	0.978-5.072	0.056	2.699	1.064-6.843	**0.036**
Differentiation	Well + moderate	1.415	0.738-2.712	0.296	1.177	0.588-2.353	0.646
FIGO stage	IB	2.321	1.218-4.423	**0.011**	2.723	1.258-5.892	**0.011**
Tumor size	<40mm	1.103	0.572-2.127	0.770	0.663	0.314-1.400	0.282
Pelvic lymph nodes resected	<25	1.281	0.585-2.804	0.535	0.893	0.394-2.024	0.785
Risk factor	Intermediate-risk	2.854	1.497-5.443	**0.001**	2.602	1.226-5.522	**0.013**
Consolidation chemotherapy	No	1.743	0.909-3.341	0.094	0.810	0.370-1.771	0.597

The bold values mean p value less than 0.05.

**Table 3:** Univariate and multivariate analysis for the overall survival of the entire cohort before propensity score matching.

Characteristics	Reference	Overall survival					
		Univariate analysis			Multivariate analysis		
		HR	95% CI	P	HR	95% CI	P
Histology	Squamous	2.037	0.774-5.359	0.150	2.929	0.980-8.758	0.054
Differentiation	Well + moderate	1.155	0.541-2.466	0.709	0.911	0.405-2.050	0.821
FIGO stage	IB	2.458	1.169-5.172	**0.018**	3.072	1.244-7.585	**0.015**
Tumor size	<40mm	1.116	0.522-2.383	0.778	0.699	0.295-1.654	0.415
Pelvic lymph nodes resected	<25	1.289	0.522-3.180	0.582	0.934	0.363-2.407	0.888
Risk factor	Intermediate-risk	2.590	1.234-5.434	**0.012**	2.513	1.031-6.129	**0.043**
Consolidation chemotherapy	No	1.552	0.739-3.262	0.246	0.674	0.266-1.706	0.405

The bold values mean p value less than 0.05.

### 3.3 Efficacy of consolidation chemotherapy after adjuvant therapy in the subgroups

**Figure 3:**
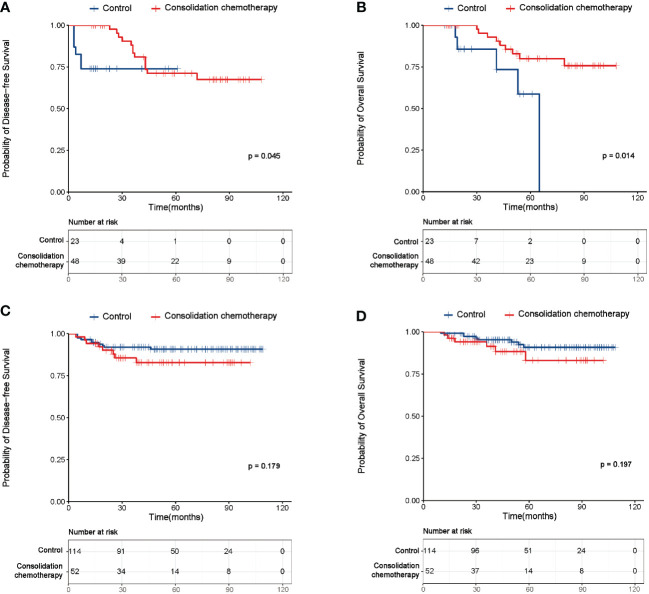
Survival curves of the study and control group of patients with high or intermediate risk factors. **(A)** Disease-free survival (DFS) curves of the study and control group of the patients with high risk factors. **(B)** Overall survival (OS) curves of the study and control group of the patients with high risk factors. **(C)** DFS curves of the study and control group of the intermediate risk factors. **(D)** OS curves of the study and control group of the intermediate risk factors.

For further investigating the actual efficacy of consolidation chemotherapy after adjuvant therapy, the subgroup analysis was firstly performed based on risk factors. A total of 71 patients were included in the subgroup with high-risk factors, where 19 (26.8%) patients experienced relapse, and 14 (19.7%) patients died during the follow-up period. The baseline characteristics of this subgroup were summarized in **Supplementary Table 3**. No significant difference was observed in the baseline characteristics between the study and control group, except for age (p=0.005). The survival analysis demonstrated the study group had a significantly better DFS and OS than the control group (DFS: p=0.045, **Figure 3A**; OS: p=0.014, **Figure 3B**). The multivariate analysis demonstrated consolidation chemotherapy after adjuvant therapy was an independent prognostic factor for both the DFS and OS of early-stage CC patients with high risk factors (**Supplementary Tables 4**, **5**).

Since a total of 61 (85.9%) patients had lymph node metastasis (LNM) in the subgroup with high risk factors, we next made efforts to investigate whether the number of LNM (nLNM) affected the benefit of consolidation chemotherapy. As shown in **Supplementary Figures 1A, B**, the study group had no better DFS or OS than the control group in patients with more than one metastatic lymph nodes (DFS: p=0.099, **Supplementary Figure 1A**; OS: p=0.054, **Supplementary Figure 1B**). However, the study group had a significantly better DFS and OS than the control group in patients with more than two metastatic lymph nodes (DFS: p=0.002, **Supplementary Figure 1C**; OS: p=0.008, **Supplementary Figure 1D**).

With regard for patients with intermediate-risk factors (n=166), recurrence was observed in 18 (10.8%) patients with 14 (8.4%) death cases during the follow-up period. The baseline characteristics of this subgroup were summarized in **Supplementary Table 6**. No significant difference was observed in age, differentiation, number of pelvic lymph nodes retrieved, LVSI and DSI between the two groups (all p>0.05). However, significant difference was observed in histologic type (p=0.001), FIGO stage (p=0.001) and tumor size (p=0.009) between the groups. As shown in **Figures 3C, D**, both the DFS and OS failed to differ between the study and control group (DFS: p=0.179; OS: p=0.197). The multivariate analysis identified histological type and FIGO stage as independent factors affecting DFS (**Supplementary Table 7**), while none of the clinical factors were found to associate with OS in patients with intermediate-risk factors (**Supplementary Table 8**).

### 3.4 Safety assessment for consolidation chemotherapy after adjuvant therapy

The details of therapy-related adverse events in the entire cohort were provided in **Table 4**. In hematologic toxicities, the study group had a significantly higher proportion of neutropenia, anemia and thrombocytopenia than the control group, regardless of any grade or ≥ grade 3 (all p<0.05). No significant difference was found in the gastrointestinal toxicities and other adverse events between the two groups, except for hepatocyte injury (study group *vs.* control group, 9.00% *vs.* 2.92%, p=0.042). For patients with high-risk factors, consolidation chemotherapy was significantly associated with a higher incidence of neutropenia and anemia, while no significant difference was found in other adverse events (**Supplementary Table 9**). For patients with intermediate-risk factors, a higher proportion of neutropenia, anemia and thrombocytopenia were also observed in the study group as compared with the control group (all p<0.05, **Supplementary Table 10**). The proportion of other adverse events did not differ between the two groups (all p>0.05).

**Table 4:** Safety assessment for consolidation chemotherapy after adjuvant therapy in the entire cohort.

Complications	Control group (n=137)	Study group (n=100)	P value
Hematologic toxicities			
Neutropenia			
Any grade	95(69.34%)	99(99.00%)	**P<0.001**
Grade ≥3	17(12.41%)	48(48.00%)	**P<0.001**
Anemia			
Any grade	97(70.80%)	100(100.00%)	**P<0.001**
Grade ≥3	4(2.92%)	23(23.00%)	**P<0.001**
Thrombocytopenia			
Any grade	64(46.72%)	67(67.00%)	**P=0.002**
Grade ≥3	5(3.65%)	13(13.00%)	**P=0.007**
Gastrointestinal toxicities			
Nausea	59(43.07%)	50(50.00%)	P=0.290
Vomiting	21(15.33%)	24(24.00%)	P=0.093
Abdominal pain	11(8.03%)	12(12.00%)	P=0.308
Diarrhea	70(51.09%)	53(53.00%)	P=0.772
Decreased appetite	61(44.52%)	55(55.00%)	P=0.111
Constipation	40(29.20%)	35(35.00%)	P=0.343
Other adverse events			
Febrile neutropenia	2(1.46%)	6(6.00%)	P=0.073
Fatigue	42(30.66%)	27(27.00%)	P=0.541
Hepatocellular injury	4(2.92%)	9(9.00%)	**P=0.042**
Renal failure	1(0.73%)	4(4.00%)	P=0.165
Small bowel obstruction	5(3.65%)	2(2.00%)	P=0.702
Infection	2(1.46%)	5(5.00%)	P=0.136

The bold values mean p value less than 0.05.

## 4 Discussion

Although radical surgery is currently the standard treatment for most early-stage CC patients, postoperative occurrence may occur partly due to insufficient radicality or potential tumor dissemination during colpotomy (13). In addition, patients with high or intermediate risk factors are found to have higher recurrence rate than others in the early-stage subpopulation (14, 15). Therefore, adjuvant radio(chemo) therapy is increasingly recommended for these patients, but its actual efficacy is still controversial and designing tailored therapeutic schedules is extremely challenging (16). A retrospective study has found adjuvant radiotherapy/chemoradiotherapy failed to provide significant benefits for OS or DFS in early-stage CC patients with one intermediate-risk factor (17). For a considerable proportion of early-stage CC patients with pelvic lymph node metastases, adjuvant radiotherapy concurrent with chemotherapy failed to provide superior survival benefits as compared with chemotherapy alone (18). In this study, we focused on a hot topic that whether consolidation chemotherapy following adjuvant therapy could be beneficial and safe for early-stage CC patients with risk factors, which might contribute to more precise therapy decision in patient management.

Firstly, our study demonstrated that consolidation chemotherapy failed to significantly improve the OS and DFS in the entire cohort, which was subsequently confirmed in the PSM cohort. This finding is inconsistent with some studies supporting its beneficial role in LACC patients. For instance, a retrospective study has found consolidation chemotherapy after cisplatin-based chemoradiotherapy resulted in longer OS, PFS and distant metastasis free survival in CC patients with FIGO stage ranging from IB2 to IVB (19). For FIGO stage IIIB/IVA patients, despite of small sample size (n=30), consolidation chemotherapy was found to effectively improve OS and PFS as compared with concurrent chemoradiotherapy and radiotherapy alone respectively (20). A single-arm retrospective study has also found consolidation chemotherapy was correlated with a remarkable local control rate (94%) and acceptable toxicities (21). However, there is also evidence suggesting not all the CC patients with FIGO stage IB, IIA and IIB could benefit from consolidation chemotherapy after postoperative concurrent chemoradiotherapy, suggesting the necessity of careful patient selection before therapy decision (22). In addition, the safety assessment demonstrated consolidation chemotherapy was associated with a higher incidence rate of hematologic toxicities and liver injury in the entire cohort, suggesting its nonnegligible detrimental impact. These adverse events were largely attributed to additional administration of chemotherapeutic agents such as paclitaxel, cisplatin and carboplatin. For instance, paclitaxel directly blocks the division cycle of hematopoietic stem cells, resulting in neutropenia (23). Cisplatin is correlated with increased incidence of hepatotoxicity, while carboplatin commonly induces thrombocytopenia (24). In the univariate and multivariate analysis, the risk factor classification is identified as an independent prognostic factor of DFS in both the entire and PSM cohort. Therefore, we hypothesized that the beneficial role of consolidation chemotherapy might differed between the high and intermediate risk group, which was then validated in the following subgroup analysis.

For early-stage CC patients with high risk factors, consolidation chemotherapy was significantly correlated with improved OS and DFS. More importantly, it was identified as an independent favorable factor affecting OS and DFS in these patients. A previous phase III randomized study (n=146) has demonstrated an improved tendency of both OS and DFS in high-risk CC patients receiving postoperative concurrent paclitaxel/cisplatin chemoradiotherapy plus consolidation chemotherapy, although the result failed to research statistical significance (25). Similar results were also observed in another study and moreover consolidation chemotherapy was proved to dramatically decrease the chance of distant metastases rather than local recurrence (8). In contrast, a small sample retrospective study (n=37) has found consolidation chemotherapy resulted in inferior 3-year OS and PFS in high-risk CC patients, as compared with survival data in other studies (26). With regard to safety assessment, higher incidence rate of hematologic toxicities was still observed in the study group as compared with the control group, which was in accordance with previous studies (8, 25). Therefore, preventive measures for hematologic toxicities are highly recommended in these patients, such as intensive blood test and recombinant human Granulocyte Colony Stimulating Factor (rhG-CSF) injection. Accumulating evidence have highlighted the crucial prognostic value of the number of lymph node metastases (nLNM) in early-stage CC patients (27, 28). We next made efforts to clarify its correlation with the benefit of consolidation chemotherapy in high-risk early-stage CC patients. As a result, consolidation chemotherapy was significantly associated with better outcome in patients with nLNM≥2 instead of those with nLNM≥1, which was in accordance with a recent study (8). This result may be partly explained by the fact that the 5-year recurrence rate was dramatically increased in early-stage CC patients with more LNM and they were more likely to benefit from consolidation chemotherapy (29). Furthermore, a previous study has demonstrated a relative high rate of distant metastasis in early-stage CC patients with LNM, supporting the feasibility of consolidation chemotherapy following adjuvant therapy (30).

The adjuvant therapies for early-stage CC patients with intermediate risk factors are still controversial, which may be partly attributed to lack of convincing evidence and high heterogeneity of this subpopulation (6). A recent nationwide retrospective study (n=6192) has suggested adjuvant therapies should be cautiously recommended in some of these subpopulation, where adjuvant chemotherapy may be effective in patients with large tumors (31). Another retrospective study has found no significant correlation between postoperative adjuvant therapies and clinical outcomes in stage IA1 to IIA2 CC patients with one intermediate-risk factor (17). However, the opposite result was observed in a study that proved stage I-IIA CC patients could benefit from postoperative chemotherapy or radiotherapy or sequential chemotherapy and radiotherapy (32). An ongoing phase III multicenter clinical trial is aimed to assess the actual efficacy of adjuvant external beam radiotherapy ± brachytherapy ± concomitant chemotherapy in these patients, and the primary result is expected to be published by 2031 (33). In this study, using a retrospective cohort enrolling 166 early-stage CC patients with intermediate-risk factors, we found consolidation chemotherapy following adjuvant therapies failed to provide more benefit of OS and DFS. The same result was also observed in a previous study with a smaller sample size (n=59), although none of patients in the study group (n=9) experienced recurrence as compared with five recurrence cases in the control group (n=50) (9). In addition, the safe assessment demonstrated a significantly higher rate of grade ≥3 hematologic toxicities in the study group as compared with the control group. Considering its efficacy and safety, consolidation chemotherapy may need to be cautiously considered in early-stage CC patients with intermediate-risk factors. On the other hand, several studies have provided more precise risk stratification in these patients, which may contribute to selecting appropriate patients for adjuvant therapies combined with consolidation chemotherapy. For instance, a multicenter study has demonstrated lymphovascular space invasion, non-squamous cell carcinoma histology, and vaginal invasion were independent adverse prognostic factors for CC patients with intermediate risk factors (34). The controlling nutritional status (CONUT), consisting of the serum albumin level, total blood cholesterol level and total peripheral lymphocyte count, has recently utilized as a predictor for using concurrent chemotherapy in early-stage CC patients with intermediate risk factors (35). Therefore, combination of traditional intermediate risk factors, other clinicopathological features and laboratory testing may be a promising approach for identifying appropriate patients who needs more intense therapies in this highly heterogeneous subpopulation.

There are some limitations in our study. Firstly, the 2009 FIGO staging was used in our study, which was revised in 2018. The outdated staging system may limit the actual impact of our findings and should be replaced with the 2018 FIGO staging in our following work. Secondly, the sample sizes of both the entire and PSM cohort were relatively small, and the actual efficacy of consolidation chemotherapy after adjuvant therapy needs to be validated in more studies with large-scale samples. Thirdly, due to the nature of retrospective work, some heterogeneous factors inevitably affected the results, such as patient selection, unbalanced baseline clinicopathological features, therapy cycles and chemotherapy regimens. For addressing this issue, well-designed multicenter clinical trials are highly encouraged. Fourthly, whether the number of high or intermediate risk factors affects the benefit of consolidation chemotherapy remains unknown and should be paid more attention in our following work. Fifthly, due to the limited sample size, our study failed to investigate the impact of consolidation chemotherapy in patients with other histological types such as neuroendocrine carcinoma and gastric-type adenocarcinoma. Finally, our safety assessment focused on the short-term adverse events and whether consolidation chemotherapy would induce some long-term complications needs to be further investigated.

In summary, consolidation chemotherapy after adjuvant therapy significantly improved both the OS and DFS of patients with high-risk factors, particularly those with nLNM≥2. However, it was associated with higher rates of grade≥3 hematologic toxicities. These findings suggest consolidation chemotherapy after adjuvant therapy should be carefully considered in early-stage CC patients with risk factors. In the future, more randomized controlled trials are still needed to further clarify its actual efficacy and safety.

## Data availability statement

The original contributions presented in the study are included in the article/**Supplementary material**. Further inquiries can be directed to the corresponding authors.

## Ethics statement

The studies involving humans were approved by ethics committee of Affiliated Hospital of Yangzhou University. The studies were conducted in accordance with the local legislation and institutional requirements. Written informed consent for participation was not required from the participants or the participants’ legal guardians/next of kin in accordance with the national legislation and institutional requirements.

## Author contributions

JXW: Conceptualization, Writing – original draft. HG: Data curation, Formal Analysis, Writing – original draft. JY: Resources, Software, Writing – review & editing. JM: Data curation, Writing – review & editing. YW: Validation, Visualization, Writing – review & editing. RG: Data curation, Writing – review & editing. XY: Funding acquisition, Writing – review & editing. JW: Conceptualization, Writing – review & editing.

## Supplementary material

Supplementary Figure 1 Survival curves of the study and control group of patients with lymph node metastasis. **(A)** Disease-free survival (DFS) curves of the study and control group of the patients with ≥1 metastatic lymph nodes. **(B)** Overall survival (OS) curves of the study and control group of the patients with ≥1 metastatic lymph nodes. **(C)** DFS curves of the study and control group of the patients with ≥2 metastatic lymph nodes. **(D)** OS curves of the study and control group of the patients with ≥2 metastatic lymph nodes.

Supplementary Table 1 Univariate and multivariate analysis for the disease-free survival of the entire cohort after propensity score matching.

Supplementary Table 2 Univariate and multivariate analysis for the overall survival of the entire cohort after propensity score matching.

Supplementary Table 3 Baseline characteristics of the high-risk cohort. LVSI, lymphovascular space involvement; LN, lymph node.

Supplementary Table 4 Univariate and multivariate analysis for the disease-free survival of the patients with high risk factors.

Supplementary Table 5 Univariate and multivariate analysis for the overall survival of the patients with high risk factors.

Supplementary Table 6 Baseline characteristics of the intermediate-risk cohort. LVSI, lymphovascular space involvement; LN, lymph node.

Supplementary Table 7 Univariate and multivariate analysis for the disease-free survival of the patients with intermediate risk factors. LVSI, lymphovascular space involvement.

Supplementary Table 8 Univariate and multivariate analysis for the overall survival of the patients with intermediate risk factors. LVSI, lymphovascular space involvement.

Supplementary Table 9 Safety assessment for consolidation chemotherapy after adjuvant therapy in the patients with high risk factors.

Supplementary Table 10 Safety assessment for consolidation chemotherapy after adjuvant therapy in the patients with intermediate risk factors.
