# Supplementary figures and images for: Efficacy and safety of consolidation chemotherapy after adjuvant therapy in stage IB-IIA cervical cancer patients with risk factors: a retrospective single-center study
Source: Front Oncol. 2024 Mar 21;14:1374195. doi: 10.3389/fonc.2024.1374195 (PMC10991694; doi:10.3389/fonc.2024.1374195)

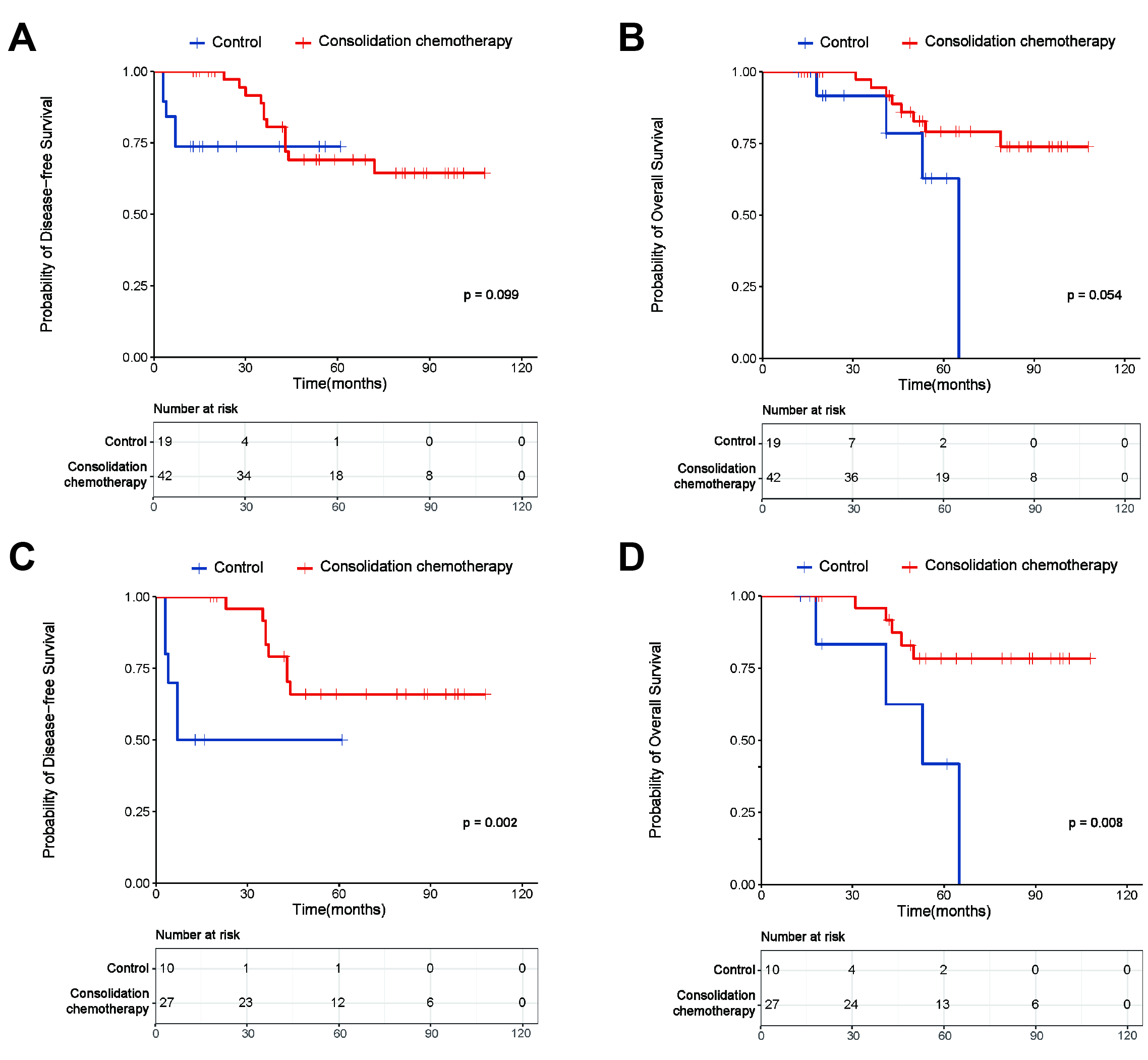

Supplement: Supplementary Figure 1 — Survival curves of the study and control group of patients with lymph node metastasis. (A) Disease-free survival (DFS) curves of the study and control group of the patients with ≥1 metastatic lymph nodes. (B) Overall survival (OS) curves of the study and control group of the patients with ≥1 metastatic lymph nodes. (C) DFS curves of the study and control group of the patients with ≥2 metastatic lymph nodes. (D) OS curves of the study and control group of the patients with ≥2 metastatic lymph nodes. [file Image_1.tif]
