# Supplementary material for: Efficacy and safety of consolidation chemotherapy after adjuvant therapy in stage IB-IIA cervical cancer patients with risk factors: a retrospective single-center study
Source: Front Oncol. 2024 Mar 21;14:1374195. doi: 10.3389/fonc.2024.1374195 (PMC10991694; doi:10.3389/fonc.2024.1374195)
Supplement: Supplementary Table 1 — Univariate and multivariate analysis for the disease-free survival of the entire cohort after propensity score matching. [file Table_1.docx]

**Supplementary Table 1. Univariate and multivariate analysis for the disease-free survival of the entire cohort after propensity score matching**

| **Characteristics** | **Reference** | **Disease-free survival** | | | | | | |
| --- | --- | --- | --- | --- | --- | --- | --- | --- |
|  |  | **Univariate analysis** | | |  | **Multivariate analysis** | | |
|  |  | **HR** | **95% CI** | **P** |  | **HR** | **95% CI** | **P** |
| Histology | Squamous | 1.632 | 0.373-7.139 | 0.515 |  | 3.998 | 0.734-21.769 | 0.109 |
| Differentiation | Well + moderate | 0.936 | 0.330-2.656 | 0.901 |  | 0.990 | 0.319-3.076 | 0.986 |
| FIGO stage | IB | 3.440 | 1.271-9.310 | **0.015** |  | 6.194 | 1.839-20.856 | **0.003** |
| Tumor size | <40mm | 0.937 | 0.356-2.465 | 0.896 |  | 0.470 | 0.142-1.549 | 0.215 |
| Pelvic lymph nodes resected | <25 | 1.172 | 0.337-4.081 | 0.803 |  | 0.790 | 0.201-3.115 | 0.737 |
| Risk factor | Intermediate-risk | 3.567 | 1.352-9.413 | **0.010** |  | 4.262 | 1.530-11.873 | **0.006** |
| Consolidation chemotherapy | No | 1.047 | 0.404-2.715 | 0.925 |  | 0.916 | 0.329-2.548 | 0.866 |

**Supplementary Table 2. Univariate and multivariate analysis for the overall survival of the entire cohort after propensity score matching**

| **Characteristics** | **Reference** | **Overall survival** | | | | | | |
| --- | --- | --- | --- | --- | --- | --- | --- | --- |
|  |  | **Univariate analysis** | | |  | **Multivariate analysis** | | |
|  |  | **HR** | **95% CI** | **P** |  | **HR** | **95% CI** | **P** |
| Histology | Squamous | 2.386 | 0.529-10.773 | 0.258 |  | 5.832 | 0.934-36.415 | 0.059 |
| Differentiation | Well + moderate | 0.918 | 0.283-2.981 | 0.887 |  | 0.702 | 0.195-2.528 | 0.588 |
| FIGO stage | IB | 3.195 | 1.038-9.831 | **0.043** |  | 5.164 | 1.230-21.685 | **0.025** |
| Tumor size | <40mm | 1.307 | 0.437-3.909 | 0.632 |  | 0.912 | 0.244-3.405 | 0.891 |
| Pelvic lymph nodes resected | <25 | 0.996 | 0.221-4.497 | 0.996 |  | 0.806 | 0.154-4.211 | 0.798 |
| Risk factor | Intermediate-risk | 3.040 | 1.016-9.091 | **0.047** |  | 3.711 | 1.156-11.915 | **0.028** |
| Consolidation chemotherapy | No | 0.934 | 0.313-2.792 | 0.903 |  | 0.760 | 0.234-2.470 | 0.649 |

**Supplementary Table 3. Baseline characteristics of the high-risk cohort**

| **Characteristics** | **Control group**  **(n=23, 100%)** | **Study group**  **(n=48, 100%)** | **P value** |
| --- | --- | --- | --- |
| Age (years old) |  |  |  |
| Median (IQR) | 59(49-64) | 50(45.3-53.8) | **0.005** |
| Histologic type |  |  |  |
| Squamous | 22(95.7) | 41(85.4) | 0.261 |
| Non-squamous | 1(4.3) | 7(14.6) |  |
| Differentiation |  |  |  |
| Well + moderate | 12(52.2) | 29(60.4) | 0.511 |
| Poor | 11(47.8) | 19(39.6) |  |
| FIGO stage |  |  |  |
| IB | 12(52.2) | 26(54.2) | 0.875 |
| IIA | 11(47.8) | 22(45.8) |  |
| Tumor size (mm) |  |  |  |
| <40 | 10(43.5) | 25(52.1) | 0.497 |
| ≥40 | 13(56.5) | 23(47.9) |  |
| LVSI | 20(87.0) | 42(87.5) | 1.000 |
| Deep stromal invasion | 21(91.3) | 45(93.8) | 0.656 |
| No. of pelvic LN retrieved |  |  |  |
| Median (IQR) | 16(13-19) | 18(14-28) | 0.148 |
| Pelvic LN metastasis | 19(82.6) | 42(87.5) | 0.718 |
| Parametrial invasion | 2(8.7) | 10(20.8) | 0.313 |
| Surgical margin involvement | 2(8.7) | 5(10.4) | 1.000 |

Abbreviations: LVSI, lymphovascular space involvement; LN, lymph node.

**Supplementary Table 4. Univariate and multivariate analysis for the disease-free survival of the patients with high risk factors**

| **Characteristics** | **Reference** | **Disease-free survival** | | | | | | |
| --- | --- | --- | --- | --- | --- | --- | --- | --- |
|  |  | **Univariate analysis** | | |  | **Multivariate analysis** | | |
|  |  | **HR** | **95% CI** | **P** |  | **HR** | **95% CI** | **P** |
| Histology | Squamous | 1.279 | 0.372-4.405 | 0.696 |  | 1.424 | 0.347-5.843 | 0.624 |
| Differentiation | Well + moderate | 1.638 | 0.664-4.038 | 0.284 |  | 2.069 | 0.724-5.913 | 0.175 |
| FIGO stage | IB | 1.560 | 0.627-3.881 | 0.339 |  | 2.790 | 0.885-8.798 | 0.080 |
| Tumor size | <40mm | 0.808 | 0.325-2.013 | 0.648 |  | 0.351 | 0.108-1.137 | 0.081 |
| Pelvic lymph nodes resected | <25 | 1.607 | 0.628-4.114 | 0.323 |  | 1.723 | 0.606-4.900 | 0.308 |
| Parametrial invasion | No | 1.030 | 0.299-3.545 | 0.963 |  | 2.777 | 0.421-18.298 | 0.288 |
| Surgical margin involvement | No | 1.439 | 0.418-4.953 | 0.564 |  | 2.492 | 0.616-10.078 | 0.200 |
| Positive lymph nodes | No | 1.349 | 0.311-5.849 | 0.690 |  | 3.653 | 0.518-25.760 | 0.194 |
| Additional chemotherapy | No | 0.362 | 0.129-1.022 | 0.055 |  | 0.252 | 0.079-0.807 | **0.020** |

**Supplementary Table 5. Univariate and multivariate analysis for the overall survival of the patients with high risk factors**

| **Characteristics** | **Reference** | **Overall survival** | | | | | | |
| --- | --- | --- | --- | --- | --- | --- | --- | --- |
|  |  | **Univariate analysis** | | |  | **Multivariate analysis** | | |
|  |  | **HR** | **95% CI** | **P** |  | **HR** | **95% CI** | **P** |
| Histology | Squamous | 1.181 | 0.263-5.302 | 0.828 |  | 1.852 | 0.320-10.734 | 0.492 |
| Differentiation | Well + moderate | 1.532 | 0.534-4.395 | 0.427 |  | 1.698 | 0.458-6.295 | 0.428 |
| FIGO stage | IB | 1.945 | 0.651-5.810 | 0.233 |  | 3.163 | 0.783-12.774 | 0.106 |
| Tumor size | <40mm | 0.690 | 0.231-2.062 | 0.507 |  | 0.335 | 0.085-1.323 | 0.119 |
| pelvic lymph nodes resected | <25 | 1.591 | 0.528-4.798 | 0.410 |  | 2.071 | 0.571-7.512 | 0.268 |
| Parametrial invasion | No | 0.918 | 0.205-4.113 | 0.911 |  | 1.637 | 0.136-19.664 | 0.698 |
| Surgical margin involvement | No | 2.098 | 0.584-7.535 | 0.256 |  | 4.277 | 0.822-22.259 | 0.084 |
| Positive lymph node | No | 0.934 | 0.208-4.195 | 0.929 |  | 1.623 | 0.189-13.946 | 0.659 |
| Additional chemotherapy | No | 0.264 | 0.085-0.823 | **0.022** |  | 0.191 | 0.049-0.742 | **0.017** |

**Supplementary Table 6. Baseline characteristics of the intermediate-risk cohort**

| **Characteristics** | **Control group**  **(n= 114, 100%)** | **Study group**  **(n=52, 100%)** | **P value** |
| --- | --- | --- | --- |
| Age (years old) |  |  |  |
| Median (IQR) | 51 (46.8-57.0) | 51 (44.3-58.0) | 0.805 |
| Histologic type |  |  |  |
| Squamous | 109 (95.6) | 41 (78.8) | **0.001** |
| Non-squamous | 5 (4.4) | 11 (21.2) |  |
| Differentiation |  |  |  |
| Well + moderate | 79 (69.3) | 33 (63.5) | 0.457 |
| Poor | 35 (30.7) | 19 (36.5) |  |
| FIGO stage |  |  |  |
| IB | 93 (81.6) | 30 (57.7) | **0.001** |
| IIA | 21 (18.4) | 22 (42.3) |  |
| Tumor size (mm) |  |  |  |
| <40 | 81 (71.1) | 26 (50.0) | **0.009** |
| ≥40 | 33 (28.9) | 26 (50.0) |  |
| No. of pelvic LN retrieved |  |  |  |
| Median (IQR) | 18 (14-20) | 17 (12.0-23.8) | 0.626 |
| LVSI | 76 (66.7) | 39 (75.0) | 0.280 |
| Deep stromal invasion | 92 (80.7) | 44 (84.6) | 0.543 |

Abbreviations: LVSI, lymphovascular space involvement; LN, lymph node.

**Supplementary Table 7. Univariate and multivariate analysis for the disease-free survival of the patients with intermediate risk factors**

| **Characteristics** | **Reference** | **Disease-free survival** | | | | | | |
| --- | --- | --- | --- | --- | --- | --- | --- | --- |
|  |  | **Univariate analysis** | | |  | **Multivariate analysis** | | |
|  |  | **HR** | **95% CI** | **P** |  | **HR** | **95% CI** | **P** |
| Histology | Squamous | 3.111 | 1.023-9.456 | **0.045** |  | 4.067 | 1.089-15.188 | **0.037** |
| Differentiation | Well + moderate | 1.013 | 0.380-2.699 | 0.980 |  | 0.948 | 0.342-2.628 | 0.918 |
| FIGO stage | IB | 2.348 | 0.927-5.951 | 0.072 |  | 3.066 | 1.043-9.011 | **0.042** |
| Tumor size | <40mm | 1.219 | 0.472-3.144 | 0.682 |  | 0.870 | 0.314-2.409 | 0.788 |
| Pelvic lymph nodes resected | <25 | 0.358 | 0.048-2.694 | 0.319 |  | 0.307 | 0.039-2.420 | 0.262 |
| LVSI | No | 0.668 | 0.259-1.723 | 0.404 |  | 0.645 | 0.237-1.753 | 0.390 |
| Deep stromal invasion | No | 1.885 | 0.433-8.202 | 0.398 |  | 1.475 | 0.318-6.845 | 0.620 |
| Additional chemotherapy | No | 1.870 | 0.737-4.744 | 0.187 |  | 1.257 | 0.448-3.525 | 0.663 |

Abbreviations: LVSI, lymphovascular space involvement.

**Supplementary Table 8. Univariate and multivariate analysis for the overall survival of the patients with intermediate risk factors**

| **Characteristics** | **Reference** | **Overall survival** | | | | | | |
| --- | --- | --- | --- | --- | --- | --- | --- | --- |
|  |  | **Univariate analysis** | | |  | **Multivariate analysis** | | |
|  |  | **HR** | **95% CI** | **P** |  | **HR** | **95% CI** | **P** |
| Histology | Squamous | 2.989 | 0.833-10.726 | 0.093 |  | 4.146 | 0.891-19.280 | 0.070 |
| Differentiation | Well + moderate | 0.783 | 0.245-2.498 | 0.679 |  | 0.725 | 0.213-2.474 | 0.608 |
| FIGO stage | IB | 2.147 | 0.744-6.198 | 0.158 |  | 2.571 | 0.706-9.368 | 0.152 |
| Tumor size | <40mm | 1.479 | 0.513-4.267 | 0.469 |  | 0.991 | 0.311-3.154 | 0.988 |
| Pelvic lymph nodes resected | <25 | 0.487 | 0.064-3.722 | 0.488 |  | 0.492 | 0.060-4.047 | 0.509 |
| LVSI | No | 0.718 | 0.240-2.143 | 0.552 |  | 0.809 | 0.255-2.564 | 0.719 |
| Deep stromal invasion | No | 3.448 | 0.450-26.396 | 0.233 |  | 2.860 | 0.342-23.907 | 0.332 |
| Additional chemotherapy | No | 1.984 | 0.686-5.734 | 0.206 |  | 1.368 | 0.415-4.511 | 0.607 |

Abbreviations: LVSI, lymphovascular space involvement.

**Supplementary Table 9. Safety assessment for consolidation chemotherapy after adjuvant therapy in the patients with high risk factors**

| **Characteristics** | **Control group**  **(n=23)** | **Study group**  **(n=48)** | **P value** |
| --- | --- | --- | --- |
| Hematologic toxicities |  |  |  |
| Neutropenia |  |  |  |
| Any grade | 16(69.56%) | 47(97.91%) | **P<0.001** |
| Grade ≥3 | 4(17.39%) | 25(52.08%) | **P=0.005** |
| Anemia |  |  |  |
| Any grade | 21(91.30%) | 48(100.00%) | **P=0.038** |
| Grade ≥3 | 1(4.35%) | 13(27.08%) | **P=0.027** |
| Thrombocytopenia |  |  |  |
| Any grade | 13(56.52%) | 32(66.67%) | P=0.406 |
| Grade ≥3 | 1(4.35%) | 7(14.58%) | P=0.261 |
| Gastrointestinal toxicities |  |  |  |
| Nausea | 13(56.52%) | 25(52.08%) | P=0.726 |
| Vomiting | 5(21.74%) | 12(25.00%) | P=0.763 |
| Abdominal pain | 3(13.04%) | 6(12.50%) | P=1.000 |
| Diarrhea | 12(52.17%) | 27(56.25%) | P=0.747 |
| Decreased appetite | 10(43.48%) | 26(54.17%) | P=0.399 |
| Constipation | 5(21.74%) | 14(29.17%) | P=0.508 |
| Other adverse events |  |  |  |
| Febrile neutropenia | 1(4.35%) | 4(8.33%) | P=1.000 |
| Fatigue | 6(26.09%) | 13(27.08%) | P=0.929 |
| Hepatocellular injury | 1(4.35%) | 4(8.33%) | P=1.000 |
| Renal failure | 1(4.35%) | 2(4.17%) | P=1.000 |
| Small bowel obstruction | 2(8.70%) | 1(2.08%) | P=0.243 |
| Infection | 0(0.00%) | 2(4.17%) | P=1.000 |

**Supplementary Table 10. Safety assessment for consolidation chemotherapy after adjuvant therapy in the patients with intermediate risk factors**

| **Characteristics** | **Control group**  **(n=114)** | **Study group**  **(n=52)** | **P value** |
| --- | --- | --- | --- |
| Hematologic toxicities |  |  |  |
| Neutropenia |  |  |  |
| Any grade | 79(69.30%) | 52(100.00%) | **P<0.001** |
| Grade ≥3 | 13(11.40%) | 23(44.23%) | **P<0.001** |
| Anemia |  |  |  |
| Any grade | 76(66.67%) | 52(100.00%) | **P<0.001** |
| Grade ≥3 | 3(2.63%) | 10(19.23%) | **P=0.001** |
| Thrombocytopenia |  |  |  |
| Any grade | 51(44.74%) | 35(67.31%) | **P=0.007** |
| Grade ≥3 | 4(3.51%) | 6(11.54%) | P=0.073 |
| Gastrointestinal toxicities |  |  |  |
| Nausea | 46(40.35%) | 25(55.77%) | P=0.351 |
| Vomiting | 16(14.04%) | 12(23.08%) | P=0.149 |
| Abdominal pain | 8(7.02%) | 6(11.54%) | P=0.372 |
| Diarrhea | 58(50.88%) | 26(50.00%) | P=0.916 |
| Decreased appetite | 51(44.74%) | 29(55.77%) | P=0.187 |
| Constipation | 35(30.70%) | 21(40.38%) | P=0.221 |
| Other adverse events |  |  |  |
| Febrile neutropenia | 1(0.88%) | 2(3.85%) | P=0.231 |
| Fatigue | 36(31.58%) | 14(26.92%) | P=0.544 |
| Hepatocellular injury | 3(2.63%) | 5(9.62%) | P=0.110 |
| Renal failure | 0(0.00%) | 2(3.85%) | P=0.097 |
| Small bowel obstruction | 3(2.63%) | 1(1.92%) | P=1.000 |
| Infection | 2(1.75%) | 3(5.77%) | P=0.178 |
